# Malaria-related knowledge and prevention practices in four neighbourhoods in and around Mumbai, India: a cross-sectional study

**DOI:** 10.1186/1475-2875-13-303

**Published:** 2014-08-07

**Authors:** Gaurav Dhawan, Nidhin Joseph, Penelope S Pekow, Christine A Rogers, Krishna C Poudel, Maria T Bulzacchelli

**Affiliations:** Department of Public Health, School of Public Health and Health Sciences, University of Massachusetts-Amherst, 715 N. Pleasant St, 01003 Amherst, MA USA

**Keywords:** Malaria, Mosquito, Awareness, Knowledge, Prevention, Bed nets, India

## Abstract

**Background:**

India accounts for the highest number of malaria cases outside of Africa. Eighty per cent of India’s population lives in malaria-risk areas, with cases increasing in urban areas. Mumbai, India, one of the most populous cities in the world, has experienced such an increase. To be successful, many malaria control efforts require community participation, which in turn depends on individuals’ knowledge and awareness of the disease. This study assessed the knowledge and prevention practices regarding malaria in residents of four different areas of Mumbai, India, around the time of a malaria outbreak and the start of a widespread awareness campaign.

**Methods:**

A cross-sectional comparative study assessed malaria-related knowledge and prevention practices in four geographically and socio-demographically distinct areas of Mumbai, India. A structured interviewer-administered questionnaire was administered to a stratified random sample of 119 households between 16 December 2010 and 30 January 2011. Participant socio-demographic characteristics, malaria knowledge, malaria prevention practices, and household environmental factors were examined overall and compared across the four areas of Mumbai.

**Results:**

Overall, respondents had excellent knowledge of the mosquito as the means of transmission of malaria, mosquito biting times and breeding sites, and fever as a symptom of malaria. However, many respondents also held misconceptions about malaria transmission and symptoms. Respondents generally knew that bed nets are an effective prevention strategy, but only 30% used them, and only 4% used insecticide-treated bed nets. Knowledge and prevention practices varied across the four areas of Mumbai.

**Conclusions:**

Although most residents know that bed nets are effective in preventing malaria, usage of bed nets is very low, and almost no residents use insecticide-treated bed nets. As the four areas of Mumbai differed in knowledge, prevention practices, and primary sources of information, malaria control campaigns should be tailored according to the knowledge gaps, practices, environments, resources, and preferences in different areas of the city, using the interpersonal and media channels most likely to reach the target audiences. Malaria control efforts involving bed nets should emphasize use of insecticide-treated bed nets.

## Background

According to the World Health Organization (WHO), each year there are over 200 million cases of malaria worldwide [1]. Of these annual cases, approximately 1.5 million occur in India [2]. Although India accounts for only a small fraction of global malaria, it is the country with the highest number of cases outside of Africa [3] and accounts for two-thirds of all cases in Southeast Asia [1]. There is major disagreement about the actual number of malaria deaths in India [4–9], however, research suggests that malaria in India is becoming more severe. India has been experiencing an increase in the proportion of *Plasmodium falciparum*, the more deadly parasite, from 44% of all confirmed cases in 2005 to 54% in 2009 [2], and it may soon reach 59% [4]. Furthermore, India is seeing more severe cases of the previously benign *Plasmodium vivax* in recent years [10, 11]. At the same time that malaria severity is increasing, India is making relatively slow progress in malaria control. Whereas many countries have seen declines in malaria cases of over 50% between 2000 and 2010, the decline in India over the same time period was only 28% [1] with no decline since 2007 [2].

Approximately 80% of India’s population lives in malaria risk areas [1]. Once considered strictly a rural problem, malaria has increased in urban areas [12, 13]. Control of urban malaria deserves special attention because the vector-breeding sites and appropriate control measures in rural and urban areas differ. Urban malaria is thought to be largely associated with construction activity and migrant workers [14]. Construction sites are thought to promote vector breeding because they are full of places that collect water. Moreover, workers from malaria-endemic areas migrate to the cities to work on construction projects, where they live in non-permanent housing structures that allow mosquitoes to enter easily.

Mumbai is one of the cities in India that has experienced a rise in malaria. With over 2,500 construction sites in the city, Mumbai experienced a huge outbreak of malaria during the 2010 monsoon season, with total cases up 55% and *P. falciparum* cases up 15% from the previous year [15]. There were nearly 80,000 cases of malaria in Mumbai between April 2010 and March 2011 [16, 17]. While malaria historically has been concentrated mostly in the north-eastern parts of India [4], since this outbreak, Mumbai’s Maharashtra state, in the western part of the country, is now the fourth worst affected state in the country [16]. With the threat of further outbreaks looming, local authorities in Mumbai have embarked on enhanced malaria control efforts.

In India, malaria control activities happen at both the national and local levels. The National Vector Borne Disease Control Programme (NVBDCP) is an umbrella programme run by the Government of India for the prevention and control of malaria and other vector-borne diseases [18]. Some key components of the NVBDCP are: 1) early case detection and prompt treatment; 2) integrated vector control with indoor residual spraying of insecticides, use of larvicides or larvivorous fish, and use of insecticide-treated bed nets; and, 3) education and communication to promote community participation [19]. Current challenges to controlling malaria in India include insecticide resistance in mosquito vectors and drug resistance of *P. falciparum* to chloroquine throughout the country [4, 14]. These developments make individual prevention practices such as the use of insecticide-treated bed nets critical, which means that without community participation, malaria control efforts will have limited success. Community participation depends in part on individuals’ knowledge and awareness of the disease [20]. After the 2010 malaria outbreak in Mumbai, the Municipal Corporation of Greater Mumbai (formerly the Bombay Municipal Corporation), the city’s governing body, embarked on a multifaceted approach to malaria control. Among other things, the Municipal Corporation of Greater Mumbai conducted an awareness campaign, placing posters on buildings across the city [21].

Despite the importance of community participation in malaria control efforts, little is known about the effectiveness of such awareness campaigns or knowledge of malaria in the country generally. A recent national study assessing knowledge about various aspects of malaria found significant variability across demographic and geographic groups in India [20]. Even in India’s malaria-endemic states, knowledge of the disease is inconsistent. A study specifically of village health workers (a group that would be expected to have better knowledge than most) in Orissa found that only 85% had good knowledge of malaria overall [22]. This study found that most village health workers knew about the cause, common symptoms and chloroquine treatment for malaria, but were not familiar with the less common symptoms of malaria or artemisinin treatments. The study also found that even though most village health workers knew that bed nets were effective, they did not all use them. In Mumbai, which has only more recently seen a surge in malaria, little is known about the awareness of and use of prevention practices by residents regarding the disease.

Measuring the population’s knowledge of malaria periodically is important for targeting awareness campaigns appropriately and evaluating the effectiveness of such campaigns. The objective of this study was to assess differences in knowledge of malaria and use of prevention practices among residents of four different areas of Mumbai.

## Methods

### Study design

A cross-sectional comparative study was used to assess malaria-related knowledge and prevention practices in four areas of Mumbai, India.

### Study population and setting

The study population is residents of four geographically and socio-demographically distinct areas in and around the city of Mumbai, Maharashtra State, in western India. One of the most populous cities in the world, Mumbai has a population of approximately 13 million (20 million in the larger metropolitan area) and a population density of over 20,000 persons per sq km [23]. The four sectors of Mumbai selected for study are defined as follows: 1) urban middle class (individuals living in apartment complexes in Dadar, South Mumbai), hereafter referred to as ‘city’; 2) immigrants (individuals living in construction sites at Dahisar, North Mumbai), hereafter referred to as ‘construction sites’; 3) rural lower class (individuals living in Panvel, a small town near Mumbai), hereafter referred to as ‘village’; and, 4) urban lower class (individuals living in Dharavi suburbs, Central Mumbai), hereafter referred to as ‘slums’.

### Participants

A stratified random sample of households was selected for inclusion in the study. Households within each of the four sectors of Mumbai were randomized using a random number generator list. Selected households were approached for participation. If no member from a selected household was willing to participate or the house was locked, the next house on the list was approached until a willing household was reached. To be included in the study, an individual had to be a member of the selected household who resided in that home for at least one year, be 18 years of age or older, and understand either Hindi or English. Written informed consent was obtained from all participants. The signature of a witness was obtained for any illiterate participants. This study was approved by the University of Massachusetts Amherst School of Public Health and Health Sciences Human Subjects Review Committee. A total of 119 households were included in the study: 30 each from the city, construction sites, and slums, and 29 from the village. Sample size was limited by time constraints; the sample of 119 households reflects the maximum number of interviews that could be completed during the six weeks available for data collection.

### Data collection

Data were collected between 16 December 2010 and 30 January 2011. Malaria-related knowledge and prevention practices were assessed using a structured interviewer-administered questionnaire, which was developed based on previous studies of malaria-related knowledge, beliefs and behaviours [24, 25]. The questionnaire was administered by a trained interviewer and took approximately 45 minutes to complete. After the interview, regardless of the malaria knowledge or prevention practices of the participant, all household members present were educated regarding malaria symptoms, effective treatment options and prevention strategies. The questionnaire collected information about participant socio-demographic characteristics, household environmental factors, malaria-related knowledge, and malaria prevention practices.

### Socio-demographic characteristics and environmental factors

Socio-demographic information collected included age, gender, education level, and occupation of individual respondents. Information about the household included the number of family members, number of years lived in Mumbai, religion, economic status, and income. Information collected about the environmental characteristics of the home included the type of construction, the presence of walls and screens, and the presence of standing water.

### Malaria-related knowledge

The questionnaire contained 47 items assessing malaria-related knowledge. Respondents were asked about the transmission of malaria, symptoms, treatments, and prevention strategies. A knowledge score was calculated by giving one point for each item answered correctly, with the range of possible scores being 0 to 47. Respondents who had ever heard of malaria were asked to indicate the sources of information they had received about malaria. Respondents were given prompts for interpersonal sources, such as a doctor, health worker, community leader, neighbour, or family member. Prompts were also given for various types of media, such as television, newspaper, official document, radio, poster, and internet.

### Prevention practices

Respondents who had ever heard about malaria were asked to indicate which, if any, of the following prevention practices or products they used: removing stagnant water, wearing long-sleeve clothing, repellent coils, liquid repellents, anti-mosquito spray, bed nets, or insecticide-treated bed nets. Respondents were also asked where they would go to receive treatment for malaria: hospital, family doctor, health centre, or other place.

### Data management and analysis

In the field, responses were recorded on standardized, paper questionnaire forms and checked for errors and completeness. Data were then entered into a Microsoft Excel 2007 spreadsheet, and summary scores were computed. Descriptive statistics, including frequency distributions, means, standard deviations, and ranges were used to describe participant socio-demographic characteristics, malaria knowledge, malaria prevention practices, household environmental factors, and malaria history. All data summaries were calculated separately for each of the four sectors and overall. Statistical significance tests were performed using Minitab 16 to compare socio-demographic characteristics and total knowledge scores across sectors. Analysis of variance (ANOVA) was used to compare continuous variables across sectors. Categorical variables were compared using the chi-square or Fisher’s Exact test as appropriate. A significance level of α = 0.05 was set for all statistical tests.

## Results

### Socio-demographic characteristics

The socio-demographic characteristics of the respondents are shown in Table 1. The age of the respondents ranged from 18 to 80 years, with a mean age of 37 years (SD = 14.7). Approximately 55% of the respondents were male. Education differed significantly by sector (p < 0.001) when dichotomized at secondary school level or less, compared to high school or above. The largest proportion (30%) of respondents were daily wage workers, followed by housewives (22%), and by business persons (19%). Occupation was significantly associated with sector (p <0.001). Almost all respondents from the city, village and slums had resided in Mumbai for over five years, while duration in Mumbai was significantly shorter in construction sites (p <0.001). Approximately 75% of respondents reported being from the ‘middle class’ or ‘higher class’. The greatest proportion (62%) of respondents earned Rs.5,000 ($100) or more per month. More than half (53%) of respondents had three or four persons in their family.

**Table 1:** **Socio-demographic characteristics of study respondents by sector**

	Total	City	Construction sites	Village	Slums	P*
	(N = 119) %	(N =30) %	(N =30) %	(N =29) %	(N =30) %	
**Age**						
Mean (SD), in years	37 (14.7)	46 (14.8)	29 (9.3)	38 (17.2)	35 (11.6)	<0.001
**Gender**						
Male	55	33	83	66	40	<0.001
Female	45	67	17	34	60	
**Education**						
≤ Secondary	73	47	93	69	84	<0.001
> Secondary	27	53	7	31	17	
**Occupation**						
Daily wage worker	30	0	87	28	7	<0.001
Housewife	22	27	0	21	40	
Business person	19	27	0	10	40	
Student	6	10	0	10	3	
Other	23	36	13	31	10	
**Duration in Mumbai**						
< 5 years	7	0	23	0	3	<0.001
≥5 years	93	100	77	100	97	
**Total family members**						
1-4 persons	53	53	60	55	43	0.621
5-13 persons	47	47	40	45	57	
**Religion**						
Hindus	87	100	77	97	77	0.005
Non- Hindus	13	0	23	3	23	
**Economic status**						
Higher class and Middle class	75	93	73	21	70	<0.001
Lower class and BPL	35	7	27	79	30	
**Total monthly income (Rs.)**						
<Rs.5000	38	10	47	55	40	0.002
≥Rs.5000	62	90	53	45	60	

*ANOVA or χ^2^, as appropriate.

### Environmental factors

Environmental characteristics of respondents’ homes tended to vary by sector (Table 2). Of all respondents who had heard of malaria, a majority (66%) lived in a home with permanent construction, including nearly all respondents from the city and slums. However, 90% of respondents from construction sites lived in temporary construction. Most houses had completed walls, but this factor varied greatly by sector, ranging from only 13% in construction sites to 100% in the city. Walls tended to be made of concrete, except in construction sites, where the walls were more often made of tin. Pools of water, which are breeding grounds for mosquitoes, were present near respondents’ houses in all sectors, but were especially prevalent near homes in construction sites. Open drains were a major problem in the slums. Only 6.7% of the respondents had mosquito screens at their homes. Construction projects near respondents’ homes were rare, except in construction sites. Forty per cent of respondents reported that fogging had been done in their area in the last two weeks, 66% reported fogging within the previous four weeks. Frequency of fogging varied by sector, with respondents in construction sites reporting more recent fogging by city officials.

**Table 2:** **Environmental characteristics of respondents’ homes by sector**

	Total	City	Construction sites	Village	Slums
	(N = 119) %	(N = 30) %	(N = 30) %	(N = 29) %	(N = 30) %
**Type of house**					
Permanent construction	66	100	10	66	90
Temporary construction	34	0	90	34	10
**Walls of the house**					
Wood	10	0	27	3	7
Concrete	66	100	20	62	87
Tin	12	0	43	0	3
Other	12	0	10	35	3
**House has completed walls**	60	100	13	48	77
**Presence of a water pool near house that can be a breeding ground for mosquitoes**	56	33	67	48	77
**Type of standing water**					
Pond	2	0	5	0	0
Pool of water	18	20	5	36	9
Puddle	13	0	15	29	0
Canal	7	0	20	0	0
Open drains	36	0	15	0	74
Did not mention	21	27	27	17	13
None	43	67	33	52	23
**Have mosquito screens at home**	7	0	3	7	17
**Construction projects common near home in last year**	22	10	60	7	10
**Last time the area was fogged by city officials**					
Do not remember/Never	15	3	7	45	7
1 week	16	13	33	0	17
1-2 weeks	24	23	10	14	47
2-4 weeks	26	47	27	10	20
4-8 weeks	7	3	17	3	3
>8 weeks	13	10	7	18	7

### Malaria-related knowledge

Overall, 95.7% of respondents had heard of malaria, but awareness of malaria differed by sector. All respondents from the city and slums, and all but one in the construction sites, had heard of malaria. In contrast, 17% of respondents in the village had not heard of malaria. Of respondents who had heard of malaria, knowledge of malaria symptoms, transmission and prevention strategies differed by sector (Table 3). When total knowledge scores were compared across sectors, city respondents scored significantly higher (mean = 40.2, SD 3.6) compared to respondents in the slums (mean = 36.4, SD 3.7), village (mean = 36.0, SD 5.2), and construction sites (mean = 34.7, SD 4.4; p = 0.001). Differences across sectors were also seen for individual knowledge items.

**Table 3:** **Knowledge about malaria, among respondents who have ever heard of malaria**

	Total	City	Construction sites	Village	Slums
	(N = 114) %	(N = 30) %	(N = 29) %	(N = 25) %	(N = 30) %
**Transmission of malaria**					
The bite of infected mosquito (Yes)	98	100	100	100	93
Drinking contaminated water (No)	25	47	17	24	13
Eating contaminated food (No)	33	53	21	40	17
Close contact with malaria patient (No)	51	70	35	60	43
**Common symptoms of malaria**					
Fever (Yes)	100	100	100	100	100
Headache (Yes)	83	87	90	72	83
Chill (Yes)	96	100	100	84	97
Sweating (Yes)	67	70	83	52	60
Vomiting (Yes)	53	57	48	48	57
Abdominal pain (Yes)	44	27	69	48	33
Itching (No)	68	87	66	60	60
**Ways to prevent and control malaria**					
Wear long sleeve clothing (Yes)	49	57	48	32	57
Sleep in bed nets without insecticide (Yes)	85	87	90	80	83
Use insecticide-treated bed nets (Yes)	82	87	93	72	73
Use mosquito repellent/coil (Yes)	68	73	52	72	77
Drain standing water from pots, etc. (Yes)	77	50	86	80	93
Trim bushes around the house (Yes)	71	97	55	76	57
Clean dark corner in the house (Yes)	90	100	86	88	87
Spray insecticide (Yes)	87	97	79	76	93
Take preventive medication (Yes)	40	57	35	20	47
Use fire/smoke to keep mosquitoes away (Yes)	69	50	79	76	73
**Biting time of mosquitoes that transmit malaria**					
Day time (No)	59	37	59	72	70
Night time (Yes)	97	100	100	92	97
Dusk (Yes)	97	100	100	92	97
Dawn (No)	76	90	55	76	83
Both day time and night time (No)	73	83	59	80	70
**Breeding sites of mosquitoes**					
Pond or lake (Yes)	70	83	52	76	70
Stagnant water (Yes)	92	100	83	96	90
Canal (Yes)	71	93	79	60	50
Dry and clean place (No)	93	100	79	100	93
**Places where mosquitoes are found**					
Bushes (Yes)	71	77	52	80	77
Domestic animal shelters (Yes)	89	90	97	88	80
Tropical forests (Yes)	64	70	52	72	63
Dark corners in the house (Yes)	92	97	83	100	90
Open spaces where sunlight reaches (No)	89	100	72	96	87
**Time of year when the risk of malaria is the highest**					
Winter (No)	43	57	24	60	47
Summer (No)	52	43	31	64	70
Rainy (Yes)	93	93	83	92	100

Percentage of people responding correctly (correct answer).

All of the respondents from the city, construction sites and village, and almost all respondents from the slums (93%), knew that a mosquito bite is the means of transmission of malaria. However, large proportions of respondents from all four sectors incorrectly believed that malaria could be contracted by drinking contaminated water, eating contaminated food, or close contact with a malaria patient. Almost all respondents knew that dusk and night-time are the biting times of malaria-carrying mosquitoes, but, again, large proportions of respondents also incorrectly believed that malaria-carrying mosquitoes bite during dawn and day-time. Almost all respondents correctly identified the rainy season as the time of year with the highest risk of malaria. However, a number of respondents incorrectly identified winter or summer as the times with the highest risk of malaria, especially in construction sites.

All respondents from the city and village, and at least 90% of respondents from the slums, knew that stagnant water is a breeding site for mosquitoes and that mosquitoes do not breed in dry, clean places. Knowledge of breeding sites was lower in construction sites, with only approximately 80% of respondents correctly identifying these places. Knowledge of other breeding sites, such as ponds, lakes and canals, was less complete and varied by sector. A large majority of respondents from all four sectors correctly identified dark corners and domestic animal shelters as places where mosquitoes are found. Most respondents also correctly identified bushes and tropical forests as places where mosquitoes are found, but this knowledge was lacking in about half of respondents in construction sites.

All respondents in all four sectors correctly identified fever as a symptom of malaria. However, knowledge of less common symptoms, like headache, sweating and vomiting, was less complete and differed by sector (Table 3). Almost all respondents knew that the blood smear examination is the method for confirming cases of malaria in humans. However, a substantial number of respondents incorrectly believed that X-rays or blood pressure measurements are used to confirm malaria cases. Almost all respondents knew that there are drugs to treat malaria. Many respondents did not know that malaria can re-occur without another mosquito bite, but this knowledge varied by sector.

A large majority of respondents in all four sectors correctly identified using bed nets, spraying insecticides and cleaning dark corners as effective strategies for preventing malaria. Fewer respondents knew that wearing long-sleeved clothing and taking preventive medication could prevent malaria. Knowledge of other strategies, such as draining standing water, trimming bushes around the house, using mosquito repellent coils, and using fire and smoke to keep mosquitoes away, varied by sector.

### Information sources

Of respondents who had ever heard of malaria, 70.2% reported receiving information about malaria from one or more sources. The likelihood of receiving information varied by sector, as did the sources of information reported (Table 4). The majority of the respondents in the city (93%) had received information regarding malaria, followed by those in the construction sites (80%) and slums (70%). Only 24% of respondents from the village had received information about malaria. Among several interpersonal sources of information, the largest proportion of respondents had received information regarding malaria from a hospital, health centre or doctor (50.0%), followed by a family member (42.5%). The hospital, health centre or doctor played a key role in the spread of information in the construction sites (66.7%) and slums (66.7%), while family members played a major role in the city (71.4%). Among media sources, television (70%) and newspapers or magazines (66.3%) are the major sources of malaria information for all respondents. Newspapers or magazines are a major source of information for the respondents in the city (100%) and village (57%). Television is a major source of information in the city (93%) and slums (90.5%). Respondents in construction sites tended to receive malaria information from newspapers or magazines more than other media sources.

**Table 4:** **Sources of information regarding malaria among respondents who had received information about malaria**

	Total	City	Construction sites	Village	Slums
	(N = 80) %	(N = 28) %	(N = 24) %	(N = 7) %	(N = 21) %
**Interpersonal sources**					
Hospital/Health centre/Doctor	50	32	67	15	67
Home (family member)	43	71	33	0	29
Neighbour	15	29	17	0	0
None	15	7	29	43	0
Self	11	25	4	0	5
Health worker	10	0	13	0	24
Community head/leader	8	4	8	0	14
Other	14	14	0	43	19
**Media sources**					
Television	70	93	33	43	91
Newspaper/Magazine	66	100	42	57	52
Books/Official document	23	11	21	14	43
Poster	20)	11	17	14	38
None	17	0	46	29	5
Radio	8	0	13)	0	14
Internet	6	14	0	0	5
Other	1	0	0	0	5

### Treatment

When asked the first place one would go in order to receive treatment for malaria, a large majority (81.5%) of respondents replied that they would go to the hospital in order to receive treatment, while 37% said that they would go to the family doctor. This trend was consistent across sectors (Table 5). In the village, health centres were also commonly mentioned as the first place to go for treatment.

**Table 5:** **Where to go for treatment for malaria among respondents who had heard of malaria**

	Total	City	Construction sites	Village	Slums
	(N = 114) %	(N = 30) %	(N = 29) %	(N = 25) %	(N = 30) %
Hospital	82	97	83	52	93
Family doctor	37	30	67	28	23
Health centre	8	0	0	28	3
Other	6	7	7	3	7

### Prevention practices

Among respondents who had heard of malaria, large differences were observed across sectors in the types of malaria prevention practices used (Table 6). The most common practice overall was removing stagnant water from in and around the house, which more than 90% of respondents reported doing. About three-quarters of respondents in the construction sites reported wearing long-sleeved clothing, compared to only about half of respondents in the other three sectors. The next most prevalent practice was using repellent coils, which was practiced about twice as much in the village (55%) and construction sites (50%), compared to the city (20%) and slums (27%). Use of liquid repellents was common in the city (66%), but not in the other three sectors. Bed nets were used by a large majority of respondents in construction sites (83%), but by none of the city respondents and less than a quarter of respondents from the village (24%) and slums (13%). Insecticide-treated bed net usage was very low and only seen in respondents of the slums (13%) and village (3.4%). Anti-mosquito spray usage was also uncommon, reported by only 33% of respondents in the city, 14% in the village, 7% in the slums, and 3% in the construction sites.

**Table 6:** **Malaria prevention practices among respondents who had heard of malaria**

	Total	City	Construction sites	Village	Slums
	(N = 114) %	(N = 30) %	(N = 29) %	(N = 25) %	(N = 30) %
Stagnant water removal in house	95	97	90	100	93
Stagnant water removal near house	91	93	73	100	97
Long-sleeved clothing	60	57	77	52	53
Repellent coils	38	20	50	55	27
Liquid repellents	32	67	10	17	33
Mosquito bed nets	30	0	83	24	13
Anti-mosquito spray	14	33	3	14	7
Insecticide-treated bed nets	4	0	0	3	13

## Discussion

This study provides baseline data on knowledge and prevention practices regarding malaria in residents of four areas of Mumbai, India, around the time of a malaria outbreak and the start of a widespread awareness campaign. Overall, respondents were generally aware of malaria. Respondents had excellent knowledge regarding the mosquito bite as the means of transmission, the biting times and breeding sites of mosquitoes, and fever as a symptom of malaria; however, there were misconceptions. Respondents showed good knowledge regarding bed nets, spraying of insecticides and cleaning dark corners as effective prevention strategies, but there is room for improvement in knowledge of other prevention methods. This finding is not surprising, as prior research has found similarly high rates of knowledge about malaria transmission in India, but with most respondents reporting some incorrect information [26]. Malaria knowledge was greatest among city respondents and poorest in respondents from the construction sites and village, which reflects the differences in socio-economic status and education level between sectors. This finding is also consistent with previous research that found better malaria-related knowledge in India to be associated with higher education level and urban residence, as compared to residence in rural areas or slums [20].

Overall, the most common malaria prevention practice used was removal of stagnant water in or near the house, with more than 90% of respondents reporting doing so. Prevention practices differed somewhat across sectors. Results suggest that respondents from construction sites were more likely to use bed nets and wear long-sleeved clothing than respondents from the other sectors, perhaps because the homes in construction sites tend to be temporary structures with incomplete walls that allow mosquitoes to enter easily, and the local government distributes free bed nets in those areas. However, even though most residents in construction sites reported using bed nets, none reported using insecticide-treated bed nets. City respondents on the other hand were more likely to use liquid repellents and mosquito sprays, perhaps due to their ease of use and the higher incomes in the city. Respondents from construction sites were less likely to remove stagnant water near the house, probably due to the persistent nature of waste-water ponds associated with construction. These differences suggest that appropriate and feasible prevention methods differ across sectors, depending on environmental factors, available resources and preferences. When asked about the last time the area was fogged by city officials, respondents within sectors gave a wider range of responses than would have been expected from residents of the same neighbourhood. This discrepancy in reports of fogging suggests that residents do not always know when city officials are fogging. Because knowledge of fogging may influence residents’ perceived need for other preventive measures, city officials should consider whether their communication with the public about fogging could be improved.

It is clear that knowledge is not the only factor influencing prevention practices. Although more than four-fifths of respondents believed that bed nets are effective in preventing malaria, less than one-third actually reported using them, and fewer than one in 20 participants reported using insecticide-treated bed nets. This finding is consistent with the study by Mishra, Satpathy and Panigrahi, [22] which found that even though most village health workers knew that bed nets were effective in preventing malaria, all did not use them. Higher usage of bed nets in the construction sites, where they are freely distributed, suggests that financial barriers might be limiting the use of this prevention strategy elsewhere. Prior research also suggests that bed nets are perceived as unaffordable [26], however, lack of use of bed nets in the city, where residents could probably afford them, suggests that other factors influence bed net use. For example, having to set up the bed nets every night might be perceived as an inconvenience, and sleeping in them might be uncomfortable because they restrict airflow, making it hot to sleep under them. Better home construction in the city, along with use of liquid repellents and anti-mosquito sprays there, could also reduce the perceived need for bed nets among city residents. Further research is needed to fully understand the barriers to using effective prevention methods and to identify ways to remove those barriers.

In order to improve knowledge or promote effective prevention practices, it is important to use channels that are most likely to reach the intended audience. This study found that, overall, hospitals, health centres or doctors were the most frequently cited interpersonal source of information about malaria. Media sources were even more important, with television being the most frequently cited media source for information about malaria, followed by newspapers or magazines. Health workers and posters were an important source of information in the slums only, suggesting that different avenues of communication may be necessary to reach residents of different areas of Mumbai. These findings highlight the need for public health professionals to work closely with television, newspaper and magazine outlets, as well as hospitals, health centres and doctors, to disseminate accurate and reliable malaria information.

A limitation of this study is that its cross-sectional design does not allow a temporal relationship to be established between Mumbai’s malaria awareness campaign and knowledge acquisition or prevention practices, and it cannot determine whether the awareness campaign has affected knowledge or prevention behaviours. However, this study provides baseline information for future studies of malaria knowledge and prevention practices in the four areas studied here. Another limitation is the potential for recall bias, which is always a possibility when relying on self-report. Because data were collected in winter, which is not the rainy season, it is possible that respondents did not accurately report their prevention practices or that some environmental factors observed did not reflect the environment during the rainy season when mosquitoes are of greatest concern. However, there is no reason to believe that any recall bias would be differential across the four sectors compared in this study, so any recall bias should not affect the interpretation of the study results. Finally, this study was limited to only four specific areas of Mumbai. Because other areas of the city were not represented in the study sample, the conclusions drawn about malaria-related knowledge and prevention practices might not apply to residents of other geographic areas of the city.

## Conclusions

Differences in malaria-related knowledge and prevention practices across sectors of Mumbai are apparent. While most individuals are aware of the disease and know the means of transmission, the most common symptoms and some prevention strategies, there are also knowledge gaps that need to be filled and widespread misconceptions that need to be corrected. Education regarding malaria is especially needed in the villages, where a sizeable proportion of residents seem unaware of the disease, and in the construction sites, where knowledge is also poorer. Malaria prevention campaigns should be tailored according to knowledge gaps, practices, environment, resources, and preferences in different areas of the city, using the interpersonal and media channels most likely to reach the target audiences. Where use of bed nets is feasible, malaria control efforts should emphasize use of insecticide-treated bed nets. Research exploring the reasons for use or non-use of various prevention practices is needed so that barriers to effective prevention campaigns other than knowledge can be identified and addressed.

## Abbreviations

NVBDCP: National vector borne disease control programme
WHO: World Health Organization.
